# TRAPPED - an insight into two sisters’ struggle to access treatment for a rare genetic disease

**DOI:** 10.1186/s13023-018-0774-x

**Published:** 2018-02-27

**Authors:** Mariam Al-Attar

**Affiliations:** Faculty of Health and Medicine, Furness College, Lancaster University, Lancaster, LA1 4YG UK

**Keywords:** Rheumatology, TRAPS, Anakinra, Autoinflammatory, Amyloidosis, Genetics, Rare, Funding, NHS, Student

## Abstract

Medical student training is largely focused on acquiring knowledge of diseases and their management, which may leave one with a naïve perception of what is achievable in practice, particularly in the field of rare diseases. Tumour Necrosis Factor Receptor Associated Periodic Syndrome (TRAPS) is a rare autoinflammatory disorder with a prevalence of one in a million. Its features include recurrent disabling episodes of high-grade fever associated with rash and arthralgia. Its rarity, combined with its somewhat vague and heterogenous clinical presentation, means that patients often suffer with TRAPS for years before they are diagnosed. Although it has a licensed treatment, Interleukin-1 blocker Anakinra, this is not currently funded by the NHS. This report provides an insight into the experiences of two sisters recently diagnosed with TRAPS, and the barriers they face preventing them from accessing the treatment they need, without which they are likely to suffer life-threatening organ failure. I have argued that the commissioning policy model for rare diseases needs reconsideration to improve access to Anakinra on a national level, and have highlighted the significant impact that clinicians can have on an individual level by being advocates for their patients.

## Introduction

As a medical student, I have often considered my future career with rose-tinted spectacles. Studying countless diseases and their management, I have imagined that when I gain those magical two letters in front of my name, I will be able to treat whichever patients come my way. I did not appreciate the extent of this naivety until I observed one particular consultation during my rheumatology attachment.

“The next patient has TRAPS,” my consultant informed me. I had no knowledge of this disease. “It’s incredibly rare,” she added, explaining that managing this patient had been extremely difficult. I presumed this was due to a lack of effective treatment, as is often the case for rare diseases. The patient, a young lady called Sophia, was called in.

It became immediately clear that this was not a “normal” clinic appointment. Instead of discussing symptoms, they talked about funding applications. Listening intently, I gathered that there *was* a treatment for TRAPS, but instead of the usual recommendations about adherence, Sophia was advised to “make it last”. I was burning with questions.

Afterwards, I found out that Sophia would soon have her treatment completely withdrawn, despite desperately needing it. This left me even more bewildered. This was not a side of medicine I had seen before. To make matters worse, her half-sister, Lucy, was in the same situation. I decided to pursue TRAPS further to understand how this could be possible.

## What is TRAPS?

Tumour Necrosis Factor Receptor Associated Periodic Syndrome (TRAPS) is an autosomal dominant auto-inflammatory disorder. It is incredibly rare, with an estimated prevalence of one in a million [1]. Left untreated, patients run the risk of amyloidosis and renal failure [2], a risk magnified by the particular TRAPS mutation the sisters possess.

Its rarity, along with its vague and varying clinical presentation, meant that the two sisters suffered with TRAPS for years before they were diagnosed. Recurrent attacks of fever, joint pain and rash led to an initial misdiagnosis of Systemic Juvenile Idiopathic Arthritis in Sophia. However, years of unsuccessful treatment left her feeling disillusioned, and she eventually stopped attending clinic appointments.

The breakthrough moment came when Sophia’s sister, Lucy, was referred to the rheumatology department during a disease flare-up. This was, in fact, the first time a rheumatologist had seen the symptoms in their active state, and the nature of the rash finally became clear. Having met Sophia previously, the consultant was the first to consider a genetic cause, allowing all the pieces of the puzzle to fit together, over a decade after it began.

This delay is a common occurrence for TRAPS patients, who often remain “enigmas” for years on end, passed between clinicians at an unsettling rate. However, this was just the beginning for the two sisters, who had unfortunately received a diagnosis with an acronym well suited to their subsequent predicament regarding access to treatment.

Unlike many rare diseases, TRAPS does have a licenced treatment. Studies have demonstrated that the IL-1 receptor blocker Anakinra reliably induces and maintains disease remission [2–4]. For Sophia and Lucy, Anakinra can truly be described as a wonder drug. However, obtaining it has been an immense struggle for all involved, exposing fundamental flaws in resource allocation for rare diseases.

## Barriers to treatment

Funding for rare diseases falls within the remit of NHS England’s Specialised Services (NESS), who publish commissioning policies allowing specialist centres to prescribe drugs and receive reimbursement [5]. Until recently, this meant that patients with TRAPS could receive Anakinra through the National Amyloidosis Centre. After April 2016, however, the policy and funding were withdrawn.

With no commissioning policy in place at the time the sisters were diagnosed, the National Amyloidosis Centre submitted several individual funding requests on their behalf. However, these requests were rejected repeatedly by NESS on spurious and inconsistent grounds, including one assertion that TRAPS is too rare, followed by another stating that it is not rare enough. Inadequate trial data was also cited as justification for rejection, despite the evidence base being strong enough for the drug to be licensed, and the rarity of the disease leaving little scope for Randomised Controlled Trials (RCTs). Adding to the inconsistency was the short-sighted nature of this funding refusal, as Anakinra costs far less than the dialysis that the sisters will almost certainly require if untreated.

Repeated rejections from NESS left the sisters and their doctors feeling helpless. Although their nearly life-long mystery had been solved, there appeared to be nothing anybody could do about it, simply because of the rarity of the diagnosis. Had they been diagnosed with Rheumatoid Arthritis, Anakinra would have been funded without question. I can scarcely imagine the sisters’ frustration.

At this point, there was, in fact, a peculiar turn of events, as the consultant’s refusal to give up and inspiring dedication to help the sisters led to a small breakthrough. Approaching the producers of Anakinra directly at a conference and ardently describing the sisters’ predicament, she convinced the pharmaceutical company to provide a “compassionate supply” of six months-worth of treatment.

For Sophia and Lucy, this was life-changing. For the first time, they were finally able to have a taste of the normal life denied to them by TRAPS. With Anakinra, their disease completely remitted. Sadly, this relief was short-lived. With no clear exit strategy for the end of the six months, the sisters continued to live in uncertainty, with the knowledge that their lifeline would soon be taken away. Adding a more sinister dimension to the situation was the knowledge of the potentially lethal consequences of discontinuing Anakinra once it had been initiated. It was towards the end of these six months that the consultation I saw took place, and it was for this reason that the sisters were advised to take their medication sparingly.

Meanwhile, in another turn of events, the treatment has allowed Sophia to be well enough to become pregnant, after unsuccessfully conceiving for over seven years due to active disease. This led to a flurry of requests to the hospital trust, who eventually agreed to fund Anakinra temporarily – for Sophia, until the end of her pregnancy, and for Lucy, until January. This brings us up to today, with these seemingly arbitrary dates looming, and no word of what will happen afterwards. With untiring commitment, the professor at the National Amyloidosis Centre has personally worked on a commissioning policy for TRAPS, hoping to get it approved in time to prevent any need for treatment discontinuation. For now, however, the sisters’ future remains uncertain.

## Improving access

This essay identifies two barriers to treatment for patients with TRAPS. The first is the difficulty making a diagnosis. One way to address this is by advocating a better approach to autoinflammatory disorders. In particular, in the case of intermittent signs and symptoms, enabling patients to initiate follow-up appointments during flares will allow for better assessments. This is a system already in place in some areas, but needs expansion and effective utilisation. Patients should also be advised to photograph intermittent signs; with modern smart phones, this simple piece of advice could have a significant impact.

The second barrier described has been that of funding. Taking a wider view, I believe that the commissioning policy model for rare diseases requires reconsideration. Rare diseases are already disadvantaged through lack of awareness and difficulty recruiting patients for trials; a fair approach to resource allocation is required to offset these issues. Resources are, of course, limited, and decisions may unfortunately be partly fuelled by politics. However, this is all the more reason to appeal for sensible, consistent and less short-sighted funding decisions, to ensure that patients do not get caught up in a political crossfire. This approach would be facilitated by further trial data, and so researchers should be encouraged to persevere in this field.

This essay also highlights the significant impact that clinicians can have on an individual level. The commitment of dedicated doctors is the only reason the sisters were ever able to obtain treatment. Until policies are changed nationally, this case should encourage doctors to persevere for their patients, however time-consuming and frustrating it may be. For the two sisters, it has resulted in some months of much needed relief. Although the future is uncertain, the relentless work of the clinicians involved means that there is hope for future funding.

## Final thoughts

I may have lost my rose-tinted spectacles, but I still struggle to comprehend the idea of telling a patient that I cannot help them simply because their disease is too rare. Denying treatment to one in a million is denying one patient too many. I have been inspired by the outstanding level of dedication shown by the doctors involved in the sisters’ case, and have realised that being a good doctor is so much more than diagnosing and prescribing. We should be advocates for our patients. This is particularly relevant for those with rare diseases, who often face greater barriers to accessing treatment, in a system making tough decisions with limited resources. Sophia and Lucy’s story has given me the drive to become involved in rare disease research, and has empowered me to go above and beyond to help my future patients. After all, no patient should ever be trapped.

## Abbreviations

NESS: NHS England Specialised Services
RCT: Randomised Controlled Trial
TRAPS: Tumour Necrosis Factor Receptor Associated Periodic Syndrome

## References

[1] Lainka E, Neudorf U, Lohse P, Timmann C, Stojanov S, Huss K (2009). Incidence of TNFRSF1A mutations in German children: epidemiological, clinical and genetic characteristics. Rheumatology (Oxford).

[2] Cantarini L, Lucherini OM, Muscari I, Frediani B, Galeazzi M, Brizi MG (2012). Tumour necrosis factor receptor-associated periodic syndrome (TRAPS): state of the art and future perspectives. Autoimmun Rev.

[3] Obici L, Meini A, Cattalini M, Chicca S, Galliani M, Donadei S (2011). Favourable and sustained response to anakinra in tumour necrosis factor receptor-associated periodic syndrome (TRAPS) with or without AA amyloidosis. Ann Rheum Dis.

[4] ter Haar NM, Oswald M, Jeyaratnam J, Anton J, Barron KS, Brogan PA (2015). Recommendations for the management of autoinflammatory diseases. Ann Rheum Dis.

[5] NHS England: NHS Commissioning Specialised Services. https://www.england.nhs.uk/commissioning/spec-services/. Accessed 17 Nov 2017.

